# Osteoma Cutis: An Adverse Event Following Tattoo Placement

**DOI:** 10.7759/cureus.4323

**Published:** 2019-03-26

**Authors:** Pallavi Basu, Christof P Erickson, Antoanella Calame, Philip R Cohen

**Affiliations:** 1 Dermatology, University of California, San Diego, USA; 2 Dermatology, Compass Dermatopathology, San Diego, USA; 3 Dermatology, San Diego Family Dermatology, National City, USA

**Keywords:** acquired, adverse, cutis, deposition, event, perforating, osteoma, tattoo

## Abstract

Osteoma cutis can occur as a primary or secondary cutaneous lesion. Isolated lesions of perforating osteoma cutis are uncommon and can present with varying clinical features. Adverse events that can occur following placement of a tattoo include benign and malignant neoplasms, dermatoses, infections, and miscellaneous complications. We present a case of a man who developed perforating osteoma cutis within a tattoo and propose that osteoma cutis be included among the list of adverse events that can occur in individuals who obtain a tattoo.

## Introduction

The acquisition of tattoos has increased in worldwide prevalence over the recent decades; the associated complications from tattoos have also risen in parallel. Typically, such complications may include allergic reactions, infections, and benign and malignant tumors [1]. Osteoma cutis, the development of bone in the dermis and/or subcutaneous fat, can occur as either a primary disorder or secondary to another condition; perforating osteoma cutis is rare [2-4]. We describe the case of a man who developed osteoma cutis in his tattoo, discuss the features of individuals with a single perforating osteoma cutis lesion, and review the potential adverse effects of tattoos.

## Case presentation

A 42-year-old Hispanic man with a history of tattoos covering 80% of his body surface area and regularly shaven legs presented for evaluation of an asymptomatic solitary leg lesion within a tattoo. Further history revealed that his leg tattoos were done in his early twenties with a touch-up three years prior. Subsequently, he developed a papule that was progressively increasing in size within the area that received additional tattoo pigment during the touch-up.

**Figure 1:**
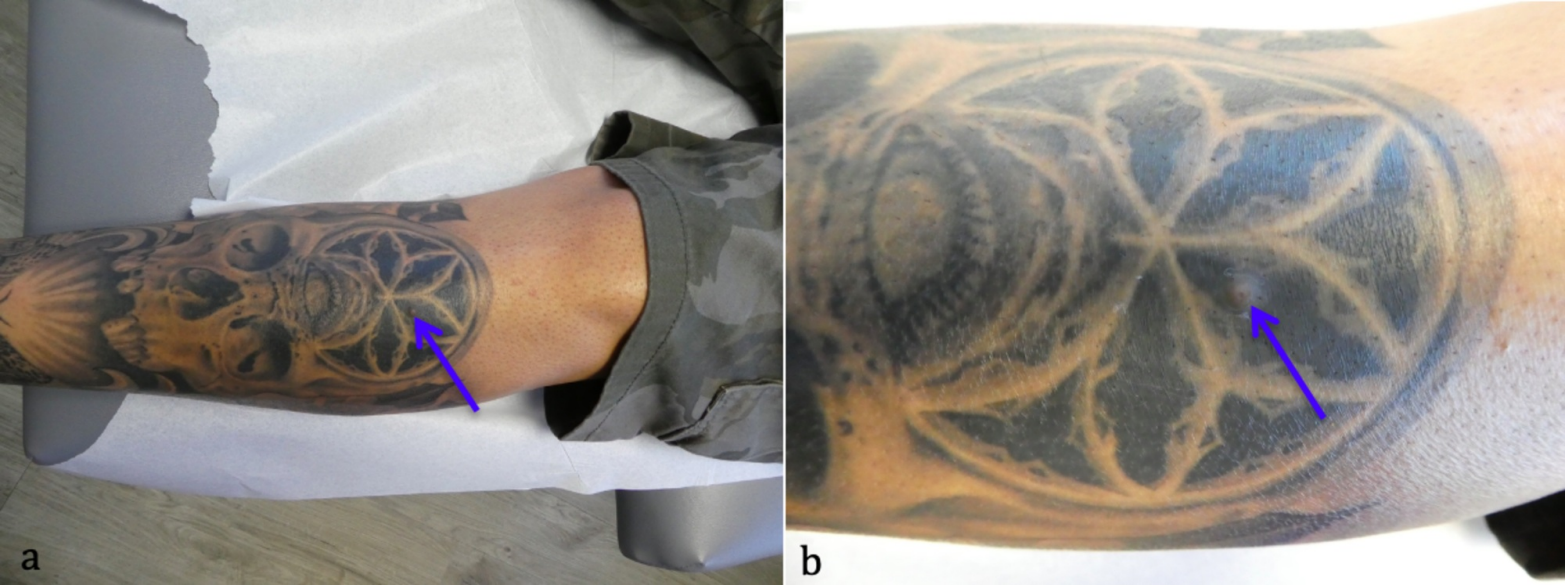
Osteoma cutis in a man with a leg tattoo Distant (a) and close-up (b) views of the left pretibial leg of a 42-year-old Hispanic man with a dermal papule with a central depression (tip of the blue arrow) that developed after he received a touch-up to his tattoo.

Cutaneous examination revealed a 5 x 5 mm dermal papule within the green tattoo pigmented area on the left pretibial leg (Figure 1). There was a depression in the center of the papule. An excisional punch biopsy was performed.

**Figure 2:**
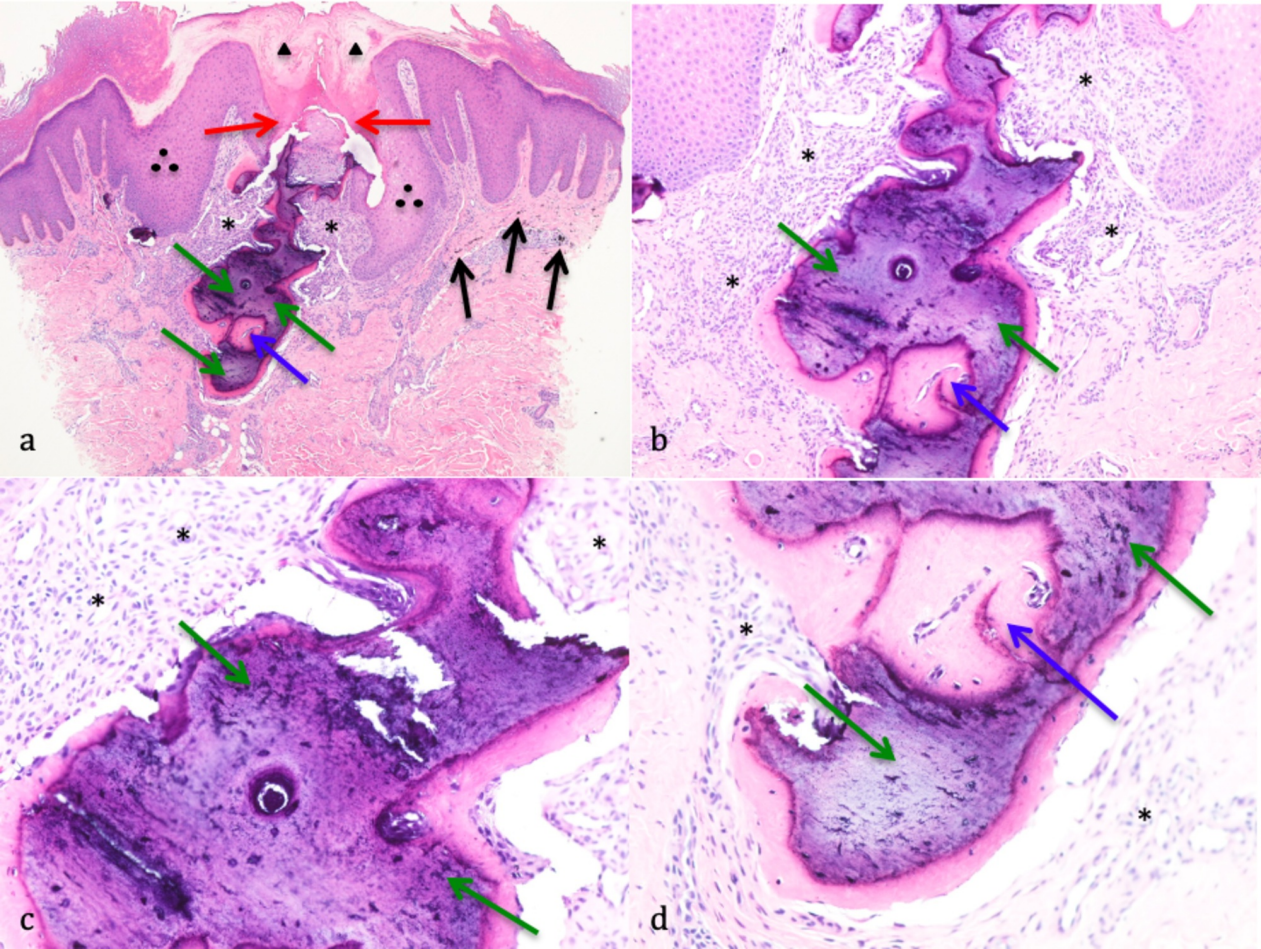
Pathology features of perforating osteoma cutis Low (a), medium (b), and high (c and d) magnification views of hematoxylin and eosin stained sections of a solitary perforating osteoma cutis lesion from the left pretibial leg of a 42-year-old Hispanic man. Bone (green arrows) with a Haversian canal (blue arrow) in the upper dermis perforates through the overlying epidermis (red arrows). The site of perforation demonstrates a keratin-plugged crater (black triangles) and extension of the adjacent hyperplastic epidermis (black circles) into the dermis. A proliferation of small vessels and a predominantly lymphocytic infiltrate (*) can be seen in the dermis surrounding the bone and below the site of perforation. Pigment from the man’s green tattoo, which appears as small black particles (black arrows), is also present in the dermis.

Microscopic examination showed bone (with Haversian canals) in the upper dermis that was perforating through the overlying epidermis. The site of perforation demonstrated a keratin-plugged crater and extension of the adjacent hyperplastic epidermis into the dermis. A proliferation of small endothelial-lined vessels and a predominantly lymphocytic inflammatory infiltrate was located in the dermis beneath the site of perforation and surrounding the bone. Also present in the dermis, adjacent to the bone, was pigment from his green tattoo which appears as small black particles (Figure 2).

He had no recurrence at six-month follow-up.

## Discussion

Osteoma cutis can be either a primary or secondary condition. It is primary in approximately 15% of patients and secondary in about 85% of patients [3,5-6]. Secondary osteoma cutis, also known as acquired osteoma cutis, typically occurs at sites of prior chronic venous insufficiency or inflammatory conditions, such as acne vulgaris, dermatomyositis, and scleroderma. It can also occur in association with trauma or cutaneous tumors, like basal cell carcinomas, chondroid syringomas, hemangiomas, nevi, and pilomatricomas [3,6-8].

The pathogenesis of osteoma cutis remains to be definitively established. In primary osteoma cutis, intramembranous ossification in the dermis may result in formation of bone [6]. One theory suggests that multiple stimuli may cause metaplasia of dermal fibroblasts and subsequent development of osteoblasts [3,9]. Another hypothesis is that bone formation occurs in response to changes in embryonic development [9].

Perforating osteoma cutis is rare. Perforating dermatoses have been observed in patients with collagenosis, elastosis, folliculitis, granuloma annulare, Kyrle disease, and renal disease [4]. Including the patient in this report, only four individuals with an isolated lesion of perforating osteoma cutis have been described (Table 1) [2-4].

**Table 1:** Features of patients with a solitary lesion of perforating osteoma cutis CR, current report; KA, keratoacanthoma; mm, millimeters; NR, not reported; y, years.

Case	1	2	3	4
Gender	Man	Woman	Man	Man
Age at diagnosis (y)	24	40	42	91
Age of onset (y)	5	20	39	90
Site	Forehead at hairline	Left breast	Left pretibial leg	Central forehead
Size (mm)	15x5	10x5	5x5	8x8
Clinical presentation	Red indurated nodule with central crust	Red nodule with keratotic central crater	Flesh-colored papule with central punctum	Flesh-colored nodule with extrusion of material from central crater
Differential diagnosis	NR	Phlebolith	Dermatofibroma, foreign body reaction to keratin, ingrown hair, seborrheic keratosis	KA, pilomatricoma, pilomatrical carcinoma, squamous cell carcinoma
Reference	2	3	CR	4

Solitary lesions of perforating osteoma cutis have been observed in three men and one woman [2-4]. Their age at diagnosis ranged from 24 years to 91 years. The location of the lesion varied: the leg in our patient, the forehead in two men [2,4], and the breast in one woman [3]. The lesions had been present for a duration ranging from less than one month to as long as 20 years prior to evaluation. One woman with a history of diabetes mellitus reported the site had thickened and drained fluid for two years prior to diagnosis [3]; all of the other patients’ lesions were asymptomatic.

The lesions ranged in size from 5 x 5 mm (our patient) to 1.5 x 0.5 cm. Two of the lesions were erythematous nodules and two were flesh-colored, including our patient. One lesion was an indurated nodule with central crusting [2], while another had a keratotic crater centrally [3], similar to our patient. Another nodular lesion had extrusion of material from the crateriform central depression [4].

Microscopically, perforation of bone through the epidermis was described for each lesion. An epidermal central channel, filled with the perforating bone, was noted; an associated inflammatory infiltrate was also present in the adjacent dermis. Bony nodules were present in the dermis and, in some lesions, calcium deposits were also apparent.

The clinical differential diagnosis was described for three of the patients. The woman, who underwent mammography of her breast lesion, was diagnosed with a phlebolith. The pathologic differential diagnosis of the older man’s lesion, from most to least likely, included keratoacanthoma, squamous cell carcinoma, pilomatricoma, and pilomatrical carcinoma. The clinical differential diagnosis for our patient’s leg lesion included an ingrown hair, a foreign body reaction to keratin, a dermatofibroma, and a seborrheic keratosis.

Treatment of the solitary perforating osteoma cutis lesion was surgical removal. One man’s lesion was diagnosed with punch biopsy followed by excision of the residual osteoma cutis [2]. Another man’s lesion was removed in its entirety with diagnostic shave biopsy [4]. In two patients, including our patient, the excisional biopsy was both diagnostic and therapeutic [3].

One patient’s perforating osteoma cutis was considered to be primary [2]. The other patients, including ours, were likely secondary. The woman’s lesion was thought to be due to superficial inflammation from chronic irritation by her brassiere [3]. One man’s lesion had appeared shortly following treatment with cryotherapy using liquid nitrogen [4]. Our patient’s lesion occurred shortly after his tattoo was touched up; to the best of our knowledge, this is the only reported individual with osteoma cutis within a tattoo.

Tattoos inks usually consist of a complex mix of organic and/or inorganic pigments dissolved in water. Issues of potential concern regarding tattoo inks include the presence of potentially toxic polycyclic aromatic hydrocarbons, organic colorants that have never been tested for use in contact with the human body, heavy metallic salts as contaminants, and the inclusion of nanoparticles [10-11]. The frequency of reported skin complications associated with tattooing varies from 2% to 27%. Approximately one-third of tattooed individuals report minor symptoms, such as itch or swelling, beyond three months after receiving their tattoo [1]. Various adverse events associated with tattoos are listed in Table 2 [1,12].

**Table 2:** Adverse events occurring within tattoos BCC, basal cell carcinoma; DFSP, dermatofibrosarcoma protuberans; KA, keratoacanthoma; MRSA, methicillin-resistant Staphylococcus aureus; PEH, pseudoepitheliomatous hyperplasia; SCC, squamous cell carcinoma. ^a^Primary dermatosis: condition primarily of skin ^b^Secondary dermatosis: systemic condition with skin involvement

Benign and malignant neoplasms	Dermatoses	Infections	Miscellaneous
Benign:	Primary^a^:	Bacterial:	Foreign body granuloma
Dermatofibroma	Allergic contact dermatitis	Clostridium tetani	Osteoma cutis
Epidermoid cysts	Darier’s disease	MRSA	PEH
Lipoma	Granuloma annulare	Staphylococcus aureus	
Milia	Lichen planus	Streptococcus pyogenes	
Seborrheic keratosis	Lichen sclerosus		
	Perforating dermatoses	Fungal:	
Malignant:	Psoriasis	Aspergillosis	
BCC	“Tattoo blow-out”	Dermatophytosis	
DFSP	Vitiligo	Mycetoma	
KA		Sporotrichosis	
Melanoma	Secondary^b^:	Zygomycosis	
SCC	Necrobiosis lipoidica		
	Lupus erythematosus	Mycobacterial:	
	Sarcoidosis	Leprosy	
	Vasculitis	Tuberculosis	
		Parasitic:	
		Leishmaniasis	
		Protozoal:	
		Syphilis	
		Viral:	
		Hepatitis B or C	
		Herpes simplex	
		Molluscum contagiosum	
		Warts	

Acute aseptic inflammatory reactions of variable intensity with erythema, induration, and an edematous “peau d’orange” dilatation of hair follicles often develop immediately during the tattooing session [13-14]. However, these reactions are often self-limited and can be associated with acute transient lymphadenopathy of the tattoo draining area. Tattoo pigment may also spread within superficial subcutaneous fat, creating a “tattoo blow-out” that appears as a halo surrounding the tattoo; although this can be permanent, it may be treated with lasers [15].

Hypersensitivity reactions can occur in response to various substances in tattoo ink or in response to any topical agent applied during the healing phase (such as disinfectants and healing ointments) in sensitized individuals. These are classified histologically, and typically include eczematous, lymphohistiocytic, lichenoid, granulomatous, sarcoidosis-like, and pseudolymphomatous reactions [12]. Delayed hypersensitivity reactions can also occur days to years later. Red and purple/violet are the most common pigments involved, perhaps due to the use of mercury or cinnabar [16]. Any granulomatous reaction should prompt evaluation for underlying idiopathic sarcoidosis, while lichenoid reactions can be associated with lichen planus [1].

Lichen planus, psoriasis, or vitiligo can also develop within a tattoo. Patients with chronic skin disorders that are known to koebnerize have a potential risk of localization of their skin disease on a tattoo. Risk factors include active disease as well as predisposing genetic features [1,12]. Other anecdotal complications include Darier’s disease, erythema multiforme, granuloma annulare, perforating dermatosis, pyoderma gangrenosum, and vasculitis. Burns and keloids may occur if laser hair removal is done on tattooed areas. The management of dermatoses in tattooed areas is the same as the treatment of these conditions in non-tattooed areas [1].

Cutaneous infections, including cellulitis, ecthyma, erysipelas, folliculitis, furunculosis, and impetigo, may occur after tattooing. There are several potential causes of the infection: the ink itself, inadequate disinfection of the skin area to be tattooed with superinfection due to scratching and subsequent inoculation of microorganisms, lack of hygiene control measures in the tattooing process, and insufficient personal hygiene [17]. Viral diseases commonly include human papillomavirus-associated warts; however, the transmission of other viruses - such as molluscum contagiosum, herpes simplex virus, or hepatitis B and C - can occur. Bacterial infections commonly include Staphylococcus aureus, Streptococcus pyogenes; atypical mycobacterial infections, presenting with nonspecific lesions one to three weeks following the tattoo, have also been observed [12,17]. 

Cutaneous malignancies appearing within tattoos include basal cell carcinoma, dermatofibrosarcoma protuberans, keratoacanthoma, melanoma, and squamous cell carcinoma. However, their discovery within tattoos may be a coincidental finding [1,18]. Surveillance of a nevus is more challenging in patients with tattoos. Indeed, the delayed detection of the development of a melanoma within a tattoo may prevent early diagnosis and management. Tattooing over a benign nevus may lead to sudden clinical changes in the pigmented lesion, warranting removal and pathologic evaluation to distinguish between trauma and malignant transformation. Similarly, distinguishing pseudoepitheliomatous hyperplasia from keratoacanthoma and squamous cell carcinoma within a tattoo can be difficult and may require histologic evaluation [1].

Miscellaneous adverse events that have been observed within tattoos include foreign body granuloma and pseudoepitheliomatous hyperplasia. To this list, we also add osteoma cutis. Indeed, to the best of our knowledge, osteoma cutis secondary to tattooing has not been previously described.

## Conclusions

Isolated lesions of perforating osteoma cutis have rarely been described and can occur as either a primary or secondary condition. Multiple types of adverse events may occur in patients with tattoos; however, to the best of our knowledge, osteoma cutis developing in a tattoo has not been previously described. Therefore, we add osteoma cutis to the list of potential complications that can occur following the placement of a tattoo.

## References

[1] Kluger N (2016). Cutaneous and systemic complications associated with tattooing. Presse Med.

[2] Hong SH, Kang HY (2003). Case of perforating osteoma cutis. Ann Dermatol.

[3] Kim BK, Ahn SK (2015). Acquired perforating osteoma cutis. Ann Dermatol.

[4] Cohen PR (2018). Perforating osteoma cutis: case report and literature review of patients with a solitary perforating osteoma cutis lesion. Dermatol Online J.

[5] Grandhe N, Dogra S, Saikia U, Handa S (2004). Acquired perforating primary osteoma cutis. Acta Derm Venereol.

[6] Moreira Amorim G, Mastrangelo Marinho Falcão EMMF, Carvalho Quintella D, Cuzzi T, Canedo de Magalhães T, Raso Bastos P (2017). Primary isolated osteoma cutis of the face. Dermatol Online J.

[7] Sanmartín O, Alegre V, Martinez-Aparicio A, Botella-Estrada R, Aliaga A (1993). Congenital platelike osteoma cutis: case report and review of the literature. Pediatr Dermatol.

[8] Fazeli P, Harvell J, Jacobs MB (1999). Osteoma cutis (cutaneous ossification). West J Med.

[9] Haro R, Revelles JM, Angulo J, Fariña MDC, Martín L, Requena L (2009). Plaque-like osteoma cutis with transepidermal elimination. J Cutan Pathol.

[10] Laux P, Tralau T, Tentschert J (2016). A medical-toxicological view of tattooing. Lancet.

[11] Dirks M (2015). Making innovative tattoo ink products with improved safety: possible and impossible ingredients in practical usage. Curr Probl Dermatol.

[12] Wenzel SM, Rittmann I, Landthaler M, Bäumler W (2013). Adverse reactions after tattooing: review of the literature and comparison to results of a survey. Dermatology.

[13] Kluger N (2012). Acute complications of tattooing presenting in the ED. Am J Emerg Med.

[14] Kluger N, Plantier F, Moguelet P, Fraitag S (2011). Tattoos: natural history and histopathology of cutaneous reactions [Article in English, French]. Ann Dermatol Venereol.

[15] Kluger N (2014). Blurry halos around tattoos: a new case of "tattoo blow-out". Int J Dermatol.

[16] Mortimer NJ, Chave TA, Johnston GA (2003). Red tattoo reactions. Clin Exp Dermatol.

[17] Long GE, Rickman LS (1994). Infectious complications of tattoos. Clin Infect Dis.

[18] Kluger N, Koljonen V (2012). Tattoos, inks, and cancer. Lancet Oncol.

